# Autoimmune Hemolytic Anemia as a Complication of Congenital Anemias. A Case Series and Review of the Literature

**DOI:** 10.3390/jcm10153439

**Published:** 2021-08-02

**Authors:** Irene Motta, Juri Giannotta, Marta Ferraresi, Kordelia Barbullushi, Nicoletta Revelli, Giovanna Graziadei, Wilma Barcellini, Bruno Fattizzo

**Affiliations:** 1 General Medicine Unit, Rare Diseases Center, Fondazione IRCCS Ca’ Granda Ospedale Maggiore Policlinico, 20122 Milan, Italy; marta.ferraresi@unimi.it (M.F.); giovanna.GRAZIADEI@policlinico.mi.it (G.G.); 2 Department of Clinical Sciences and Community Health, Università degli Studi di Milano, 20122 Milan, Italy; 3 Hematology Unit, Fondazione IRCCS Ca’ Granda Ospedale Maggiore Policlinico, 20122 Milan, Italy; jurigiann@gmail.com (J.G.); kordelia.barbullushi@unimi.it (K.B.); wilma.barcellini@policlinico.mi.it (W.B.); bruno.fattizzo@unimi.it (B.F.); 4 University of Milan, 20122 Milan, Italy; 5 Laboratorio di Immunoematologia di Riferimento, Dipartimento di Medicina Trasfusionale ed Ematologia, Fondazione IRCCS Ca’ Granda Ospedale Maggiore Policlinico, 20122 Milan, Italy; nicoletta.revelli@policlinico.mi.it; 6 Department of Oncology and Oncohematology, University of Milan, 20122 Milan, Italy

**Keywords:** autoimmune hemolytic anemia, alloimmunization, thalassemia, sickle cell disease, congenital hemolytic anemias

## Abstract

Congenital anemias may be complicated by immune-mediated hemolytic crisis. Alloantibodies are usually seen in chronically transfused patients, and autoantibodies have also been described, although they are rarely associated with overt autoimmune hemolytic anemia (AIHA), a serious and potentially life-threatening complication. Given the lack of data on the AIHA diagnosis and management in congenital anemias, we retrospectively evaluated all clinically relevant AIHA cases occurring at a referral center for AIHA, hemoglobinopathies, and chronic hemolytic anemias, focusing on clinical management and outcome. In our cohort, AIHA had a prevalence of 1% (14/1410 patients). The majority were warm AIHA. Possible triggers were recent transfusion, infection, pregnancy, and surgery. All the patients received steroid therapy as the first line, and about 25% required further treatment, including rituximab, azathioprine, intravenous immunoglobulins, and cyclophosphamide. Transfusion support was required in 57% of the patients with non-transfusion-dependent anemia, and recombinant human erythropoietin was safely administered in one third of the patients. AIHA in congenital anemias may be challenging both from a diagnostic and a therapeutic point of view. A proper evaluation of hemolytic markers, bone marrow compensation, and assessment of the direct antiglobulin test is mandatory.

## 1. Introduction

Congenital anemias include a broad spectrum of rare red blood cell (RBC) disorders classified according to the affected RBC structure. They include hemoglobinopathies, namely sickle cell disease (SCD) and thalassemia syndromes, which are by far the most prevalent [1,2], and congenital hemolytic anemias (CHAs). In SCD, the abnormal hemoglobin (Hb), called hemoglobin S (HbS), tends to form polymers in erythrocytes that deform the structure of RBC [3]. Subsequent intravascular sickling results in hemolytic anemia and recurrent occlusion of small vessels leading to vaso-occlusive crisis. In β-thalassemia, the precipitation of α-chains aggregates in erythroid precursors leads to ineffective erythropoiesis in the bone marrow and peripheral hemolysis in the intravascular and extravascular compartments. Consequent anemia and hypoxia stimulate erythroid precursor proliferation in the medullary and extramedullary compartments [2]. CHAs are heterogeneous conditions, with either dominant, recessive, or X-linked inheritance, exhibiting a clinical course ranging from mild fully compensated anemia to chronic severe hemolysis. They include defects of erythrocyte membrane proteins, red cell enzymes, and disorders due to defective erythropoiesis.

In the most severe forms of all these disorders, transfusions and iron chelation are currently the main treatment strategy [4,5,6,7,8]. The chronic course of both hemoglobinopathies and CHAs may be complicated by the abrupt drop of Hb values due to several causes, including increased destruction/sequestration (i.e., hemolytic crisis) and reduced/inhibited erythropoiesis (i.e., aplastic crisis). The latter recognizes various triggers, particularly parvovirus B19 infection [9], while the former is mainly immune-mediated. In particular, alloantibodies (alloAbs) are usually seen in chronically transfused patients and may cause severe transfusion reactions. Autoantibodies (autoAbs) have also been described, although they are rarely associated with overt autoimmune hemolytic anemia (AIHA). Autoimmunity may occur through several mechanisms, including modification of RBC membrane antigens, molecular mimicry, hidden epitopes spreading, and innocent bystander destruction [10]. Finally, both the increased destruction and impaired erythropoiesis may coexist when the autoimmune attack is directed against erythrocyte precursors [11,12]. Given the lack of data on the AIHA diagnosis and management in congenital anemias, we retrospectively evaluated all the clinically relevant AIHA cases that occurred at our hospital, a referral center for AIHA, hemoglobinopathies, and CHAs, focusing on clinical management and outcome. A review of the available literature is also provided.

## 2. Materials and Methods

We retrospectively collected clinical, laboratory, and treatment data from electronic medical records of the patients with congenital anemias followed at Fondazione IRCCS Ca’ Granda Ospedale Maggiore Policlinico who developed AIHA over a period of 20 years, between January 1991 and December 2020. The patients belonged to a cohort of 1410 followed at the Hematology Unit and the Rare Diseases Center of Fondazione IRCCS Ca’ Granda Ospedale Maggiore Policlinico, Milan, Italy, including 410 non-transfusion-dependent thalassemia (NTDT), 260 transfusion-dependent thalassemia (TDT), 190 SCD and sickle thalassemia, and 550 CHA cases, namely hereditary spherocytosis (HS), stomatocytosis (HSt), elliptocytosis (HE), and enzymopathies (mainly including pyruvate kinase deficiency). Direct and indirect antiglobulin test (DAT and IAT) results were revised by an experienced immune hematologist, and the diagnosis of AIHA was made according to international guidelines [13]. Reticulocyte count was collected when available, and the bone marrow responsiveness index (BMRI) was calculated according to the formula “absolute reticulocyte count × patient’s Hb/normal Hb” using a cutoff of 121 to discriminate well-compensated hemolytic anemia from an ineffective response [14]. 

Response to AIHA therapy was defined as complete response (CR) (Hb > 12 g/dL and normalization of all hemolytic markers), partial response (PR) (Hb > 10 g/dL or at least 2 g/dL increase in Hb, and no transfusion requirement) [15,16], and no response (NR).

The study was approved by the ethical review committee of the coordinating center “Comitato Etico Milano Area 2” and was carried out according to the principles established by the Declaration of Helsinki. A literature review by searching for the terms “congenital anemia”, “thalassemia”, “sickle cell disease”, “hereditary spherocytosis”, “hereditary elliptocytosis”, “autoimmune hemolytic anemia”, “auto-antibodies” in indexed articles in MEDLINE via PubMed and the National Library of Medicine in the last 50 years was performed.

## 3. Results

### 3.1. AIHA in the Congenital Anemias Cohort

AIHA complicated the clinical course of 14 (1%) patients regularly followed for congenital anemias, including nine β-thalassemia (mainly non-transfusion-dependent, *n* = 7), two SCD/β-thalassemia, one SCD, one HS, and one HE patients. Table 1 shows the main clinical laboratory findings collected during the AIHA episode. The median Hb values dropped from 9.5 g/dL to 5.9 g/dL during the AIHA crisis, with a significant increase in LDH. Reticulocyte response (i.e., BMRI > 121) was adequate to Hb levels in 7/11 (64%) of the evaluated subjects. AIHA cases were classified as warm (*n* = 11, of whom four were fixing complement), cold (*n* = 2), and DAT-negative (*n* = 1, patient #4, diagnosed after the exclusion of other causes of hemolysis and steroid response). Of note, eight patients exhibited anti-RBC alloAbs along with autoAbs. We were not able to identify a trigger in all the events; however, possible triggers were recent transfusion (*n* = 5), infection (*n* = 3), pregnancy (*n* = 2), and surgery (*n* = 1). All the patients received steroid therapy as the first-line, but the second- and third-line treatments were necessary in four (28.5%) and two (14%) patients, respectively, and included rituximab (*n* = 2), azathioprine (*n* = 2), intravenous immunoglobulins (*n* = 1), and cyclophosphamide (*n* = 1). Transfusion support was required in eight patients (57%) with non-transfusion-dependent congenital anemia, and recombinant human erythropoietin (rhEPO) was safely administered in five patients (36%). Hydroxycarbamide (HU) for the underlying condition was given to four patients during the acute event to obtain a reduction in transfusion requirements, while one was already on chronic treatment. On the whole, four CR, seven PR, and three NR were registered, with the median time to response of 2.5 months (range, 0.5–11). Notably, one HE (#2) and two TDT (patients ##3 and 5) subjects experienced more than one AIHA episode, and one patient (#14) died during acute AIHA because of underlying liver failure.

### 3.2. Literature Review

The data available in the literature on RBC autoAb/AIHA occurrence in congenital anemias are summarized in Table 2 and Table 3 and mainly derive from retrospective studies, case series, or case reports on thalassemia and SCD. Most studies are focused on alloAbs in transfused patients and report the presence of autoAbs in this context. Notably, all the reports except for one in β-thalassemia [17] evaluated the frequency of autoAbs rather than that of AIHA. Finally, only one study from our group evaluated the presence of autoAbs in HS, by using the more sensitive mitogen-stimulated DAT [11,18].

Overall, autoAb frequency ranges from 1% to 28.2% in β-thalassemia [17,19,20,21,22,23,24,25,26], from 0.8% to 42% in SCD [27,28,29,30], and reaches 61% in HS (likely due to more sensitive technique) [11]. Few data are available about the Ab type, with warm antibodies (IgG+) being reported in half of the DAT-positive cases, IgG+ complement (C)—in approximately one third of cases, and C+—in the remaining cases. Studies are consistent in identifying alloAbs, transfusion exposure, and splenectomy as risk factors for the development of autoAbs. Interestingly, only two observational studies and a few case reports evaluated clinically relevant AIHA, with the prevalence ranging from 1.8% to 6.4% [17,28]. The majority of autoAbs were warm, with or without complement fixation. A recent longitudinal study by Khaled et al. showed that 25 subjects developed AIHA among 385 β-thalassemia pediatric patients [17]. All the patients were transfusion-dependent, and the frequency of AIHA was inversely proportional to the number of blood transfusions received. AIHA was triggered by vaccination in two patients and by *Mycoplasma pneumoniae* infection in another. This study also showed that splenectomy in DAT-positive subjects was associated with an increased risk of AIHA in thalassemic patients. Finally, considering the autoAb type, thalassemic patients with IgG+ and C+ DAT were at higher risk for clinically overt AIHA. Overall, most AIHA patients presented with severe anemia and all required therapy. First-line steroids were the most frequent strategy, associated with IVIG in some cases. The use of various cytotoxic/immunosuppressive drugs is described for refractory cases, including azathioprine, mycophenolate mofetil, cyclophosphamide, methotrexate, vincristine, anti-thymocyte globulin, and actinomycin D. Splenectomy was performed for AIHA in selected refractory cases [17,31,32]. The use of rhEPO has been reported only in one retrospective study [28], and only one case of thalassemia with AIHA was successfully treated with rituximab [33]. Responses to various therapies are difficult to establish given the heterogeneity of treatments and the small number of patients in each report. Finally, across the various reports, two pediatric patients had a fatal outcome. These were one SCD case with both autoAbs and alloAbs who developed an over-hemolytic transfusion reaction [29] and one β-thalassemia case who had received multiple lines of immunosuppressive therapies for AIHA (prednisone, cyclophosphamide, methotrexate, vincristine, anti-human lymphocyte globulin, and actinomycin D; splenectomy) and died due to infection [32].

## 4. Discussion

Although rare, AIHA is a serious and potentially life-threatening complication of congenital anemias. Our study represents the largest cohort of congenital anemias in which the prevalence and treatment of AIHA are evaluated. In our analysis, AIHA had a prevalence of 1%, which is lower than that reported in the paper by Khaled et al. [17], which, however, included pediatric thalassemic patients only. Regarding triggers, we also observed that previous splenectomy, recent transfusions, infections, and pregnancy may be associated with the development of anti-RBC autoimmunity. However, in more than half of the patients, the trigger was not identified. Different prevalence between studies can be related either to heterogeneous populations or frequency of potential triggers. Indeed, infections are more frequent in children and in certain regions, and transfusion strategies and blood product may differ. Additionally, more severe CHAs with early transfusion requirement during childhood may be at higher risk of allo- and autoimmunization. Finally, it is largely accepted that immune system maturation is a dynamic concept evolving along with age and that the type and severity of several autoimmune conditions consistently vary from infancy to adulthood and elderly age. More importantly, in our study, we evaluated the prevalence of clinically overt AIHA, whilst most reports deal with the presence of anti-RBC autoAbs only (DAT positivity). As a matter of fact, the diagnosis of AIHA may be challenging in chronic hemolytic patients and, even more, in those on transfusions. In the former, hemolytic features are already present, and AIHA should be suspected in case of sudden drop of Hb levels or significant worsening of hemolytic markers. In the latter, a further flag may be the decrease of pretransfusion hemoglobin or the increase in the transfusion need. In addition, most patients in our series also exhibited anti-RBC alloAbs, which is a known finding in congenital anemias, particularly in transfusion-dependent ones. One of the key points is the distinction of autoAbs from alloAbs either in transfusion-dependent or non-transfusion-dependent conditions. In fact, besides the importance of assigning the best-matched RBC units to the patient, recognition of the “true” AIHA is pivotal for proper therapy. In this study, all the patients received steroids, and mostly responded, whilst about 25% required further treatment. Of note, about 1/3 of the patients received rhEPO, which has been shown to be effective in primary AIHA [37] avoiding possible myelotoxicity of immunosuppressants. Generally, therapy of AIHA in the context of congenital anemias is not codified, and therefore the guidelines available for primary forms are applied. If steroids are given cautiously in patients with congenital anemias and several comorbidities, second-line therapies raise even more concerns. Hydroxyurea, which is extensively used in SCD and some NTDT patients to increase the total amount of hemoglobin and fetal hemoglobin, has been recently demonstrated to alleviate complement activation in sickle cell patients, thus acquiring a potential role in AIHA management [38]. Use of plasma exchange (PEX) has also been reported in very severe AIHA [39] in case of steroids/IVIG refractoriness. No cases of CHA-related AIHA receiving PEX have been reported; however, its use in CHA should be carefully evaluated, especially in SCD with the risk related to increased viscosity.

In this regard, a close collaboration with a transfusion medicine specialist is fundamental to perform DAT with more sensitive methods (microcolumn and solid phase tests, washings with low ionic strength solutions, or experimental methods) [10], as well as to characterize the alloAbs by studying the eluate and performing extended phenotyping and genotyping when required. 

The pathophysiology underlying the development of anti-RBC autoAbs in congenital anemias is object of several hypotheses. Some mechanisms include the exposure to foreign antigens, as occurs in transfusion-dependent patients developing alloAbs, or during pregnancy, the molecular mimicry after infections, the spread of hidden epitopes during the hemolytic process through the deformation of erythrocyte membranes due to exposure to neoantigens, and the release of the free heme. The latter is involved in post-translational diversification of circulating Abs, may induce complement activation on SCD RBCs, thus increasing the risk of autoimmune reactions [40]. Additionally, the abnormal structure of SCD and thalassemic RBCs (prematurely expressing senescence antigens) may be more easily recognized by the immune system, as also shown by the increased amounts of RBC-bound IgG detected in these patients. Finally, the ineffective erythropoiesis typical of hemoglobinopathies, along with a proinflammatory bone marrow milieu, may also favor the development of anti-erythroblast autoAbs. 

## 5. Conclusions

In conclusion, AIHA in the context of congenital anemias may be challenging both from a diagnostic and a therapeutic point of view. Clinical suspicion should be high and prompt a proper evaluation of hemolytic markers, bone marrow compensation, and assessment of the DAT positivity for alloAbs and autoAbs.

## Figures and Tables

**Table 1:** Characteristics and clinical management of the patients with congenital hemolytic anemias (CHAs) experiencing autoimmune hemolytic anemia (AIHA) referred to our institution.

ID	Age (Years)/ Sex Category	Underlying CHA, Spleen status	Usual Hb Values (g/dL)	Year of AIHA Occurrence	Trigger	Hb Nadir during AIHA (g/dL)	LDH (U/L) (xULN), UB (mg/dL)	ARC (×10^9^/L), BMRI	sEPO (U/L)	DAT Positivity	Treatments	Response at Last Crisis, Time to Restore Usual Hb Values
1	26/F	HS	9	2018	-	6.3	430 (2), 4.3	230; 104	50	C	IV mPDN 1 mg/kg/day, folic acid, vitamin B12, oral iron	PR, 15 days
2	81/M	HE	11	2008, 2009, 2010	-	7.3	530 (2), 1.74	310; 141	27.3	IgG	IV mPDN 500 mg for 2 days, then 2 mg/kg/day, rhEPO, folic acid	PR, 1.5 months
3	50/F	TDT, splenectomy	8	1991, 2003, 2008	-	NA	NA	NA	NA	IgG	Oral PDN, azathioprine, cyclophosphamide	PR, NA
4	30/F	NTDT	10	2007	Pregnancy	4	NA	NA	NA	NA, alloAb+	IV mPDN, rhEPO, transfusions (HU for NTDT)	PR, >3 months
5	50/M	TDT	10	2009, 2012	-	NA	NA	NA	NA	IgG	Oral PDN, azathioprine	CR, NA
6	49/M	NTDT splenectomy	9.5	2013	-	7.6	559 (2.5), 2.46	396; 188	NA	IgG+C, AlloAb+	Oral PDN 5 mg/day, folic acid (HU for NTDT)	CR, 11 months
7	33/F	NTDT	9.5	2018	Pregnancy and transfusion	5.1	599 (2), 1.73	439; 160	96.8	IgG+C, alloAb+	IV mPDN 1 mg/kg/day, folic acid, transfusions	PR, 2 months
8	43/F	NTDT, splenectomy	12	2018	Transfusion, pielonephritis	6.3	510 (2), 0.83	392; 174	NA	IgG+C, alloAb+	IV mPDN 1.5 mg/kg/day, rituximab 375 mg/s.m., folic acid, vitamin B12 (HU for NTDT)	PR, >3 months
9	67/M	β-thal trait	9.5	2019	-	6.9	227 (1.06), 1.28	7; 3	173	IgG, alloAb+	Oral PDN 0.6 mg/kg/day, rhEPO, folic acid, vitamin B12, transfusions	NR
10	24/F	NTDT	8.5	2019	-	5.6	601 (2.8), 4.31	246; 98	NA	IgG, alloAb+	Oral PDN 0.5 mg/kg/day, folic acid, transfusions	NR
11	54/M	NTDT	9.5	2019	-	2.3	1378 (6.4), 4.06	30; 4	NA	C, alloAb+	IV mPDN 1.5 mg/kg/day + high-dose boli 1 g/day for 3 days, IVIG, rituximab 375 mg/s.m., rhEPO, folic acid, vitamin B12, transfusions (HU for NTDT, imiglucerase for β-glucocerebrosidase deficiency)	CR, 4 months
12	34/F	SCD	10.5	2020	Surgery, infection, transfusion	4.5	4696 (21.9), 3.33	436; 140	3365	IgG+C, alloAb+	IV mPDN 1 mg/kg/day, folic acid, vitamin B12, transfusions	CR, 2.5 months
13	58/F	SCD/β-thal, splenectomy	8	2020	Start of chronic transfusion support	6.3	1235 (5.7), 1.67	668; 301	NA	IgG	IV mPDN 1.5 mg/kg/day, rhEPO, folic acid, transfusions (HU for SCD/thal)	PR, 20 days
14	55/M	SCD/ β-thal	8.5	2020	Infection, transfusion	5	791 (3.7), 33	557; 174	130	IgG	IV mPDN 1 mg/kg/day, folic acid, transfusions	NR, dead
Summary median, range	49.5 24–81	-	9.5 8–12	-	-	5.9 2.3–7.6	599, 227–4696 2.46 0.83–33	392 7–668	113.4 27.3–3365	-	-	-

HS: hereditary spherocytosis, HE: hereditary elliptocytosis, TDT: transfusion-dependent thalassemia, NTDT: non-transfusion-dependent thalassemia, SCD: sickle cell disease, Hb: hemoglobin, LDH: lactate dehydrogenase, ULN: upper limit of normality, UB: unconjugated bilirubin, ARC: absolute reticulocyte count, sEPO: serum erythropoietin, DAT: direct antiglobulin test, C: complement, AlloAb: alloantibodies, IV: intravenous, mPDN: methylprednisolone, PR: partial response, rhEPO: recombinant human erythropoietin, NR: non-response, HU: hydroxycarbamide, PDN: prednisone, CR: complete response, IVIG: intravenous immunoglobulins.

**Table 2:** Studies and case series/reports about RBC autoAbs/AIHA in thalassemia.

Type of Study; Objective of the Study	Number of pts; Pediatric/Adult; Sex	Main Findings
Longitudinal study; to identify predictive factors of AIHA in patients with RBC autoAbs [17]	385; pediatric; F 57.5%, M 42.5%	- Frequency of RBC autoAbs: 22.6% - AIHA: 6.4% of the entire population, 28.7% of the subjects with RBC autoAbs - DAT: IgG+ 65.5%, IgG+ C+ 32.1%, IgM+ 0.2% - AIHA risk factor: prior alloimmunization, β-TI, splenectomy, the first 72 transfusions, family history of AIHA, presence of polyspecific autoAbs, AB blood type - AIHA protective factor: transfusion with phenotypic and leukoreduced blood - First-line treatment: prednisone 2 mg/kg/d - Second-line treatments: IV methylprednisolone 1000 mg/m^2^/day for three days (*n* = 5), azathioprine (*n* = 7), mycophenolate mofetil (*n* = 4), splenectomy (*n* = 2)
Prospective observational study; incidence of RBC autoAbs [19]	500; pediatric and adult; F 57%, M 43%	- Frequency of RBC autoAbs: 1% - No association with alloimmunization
Cross-sectional study [20]	407; pediatric and adult; F 55%, M 45%	- Frequency of RBC autoAbs: 6.5% - Risk factor: alloAbs
Frequency of RBC autoAbs [21]	301; NA; NA	- Frequency of RBC autoAbs: 15.9% - DAT: IgG+ 56.3%, IgG+ C+ 10.4%, C+ 33.3% - Risk factor: alloAbs
Frequency of RBC autoAbs [22]	319; pediatric and adult; F 26.3%, M 73.7%	- Frequency of RBC autoAbs: 28.2% - Risk factor: age, splenectomy, number of transfusions
Case-control study; prevalance of autoAbs [23]	280; pediatric and adult; F 33.3%, M 66.7%	- Frequency of RBC autoAbs: 1.8 % - DAT: IgG+ 100% - All (*n* = 5) developed clinically significant AIHA - Treatment: steroids
Frequency of RBC autoAbs [24]	200; pediatric and adult; F 53%, M 47%	- Frequency of RBC autoAbs: 16.5% - Risk factor: age, duration of transfusion support and the total number of transfusions, splenectomy
Retrospective and prospective observational study; prevalence of RBC autoAbs; risk factor analysis [25]	118; pediatric and adult; F 50%, M 50%	- Frequency of RBC autoAbs: 22.8% - 37% transient autoAbs without any treatment - DAT: IgG+ 48%, IgG+ C+ 52% - Risk factor: alloAbs, splenectomy
Frequency of RBC autoAbs [26]	49; pediatric and adult; F 49%, M 51%	- Frequency of RBC autoAbs: 2.14 %
Case series of AIHA [31]	4; pediatric; F 25%; M 75%	- DAT: IgG+ - Treatment: - Case 1: High-dose IVIG (CR) - Case 2: prednisone therapy (ineffective); then azathioprine (PR); then High-dose IVIG (CR). Splenectomy for significant splenomegaly - Case 3: prednisone therapy (ineffective); then High-dose IVIG (PR). - Case 4: prednisone therapy (PR); then High-dose IVIG (CR). Recurrence after therapy interruption: treatment with IVIG repeated, with CR - The standard dose of 2 g/kg was used for IVIG treatment
Case report of AIHA [34]	2; pediatric; F 50%, M 50%	- Both cases of AIHA presented with severe anemia - DAT: C+ (case 1); negative at diagnosis, then strongly positive (case 2) - Treatment: transfusions, IVIG 2 g/kg + methylprednisolone 30 mg/kg/d for 3 days, followed by prednisolone 2 mg/kg/d in both cases - Response: PR weeks later
Case report of AIHA [32]	1; pediatric; F	- AIHA presenting with severe anemia - DAT: initially negative, IgG+ after splenectomy - Treatment: transfusion, oral prednisone 30 mg/day, cyclophosphamide 50 mg/day, and methotrexate 2.5 mg/day: no hemolysis reduction. Treatment with vincristine 1 mg/week, anti-human lymphocyte globulin 125 mg/day IV, and actinomycin D 15 mcg/kg/day for 5 days led to transient decrease in transfusion requirement. Splenectomy (for splenomegaly): no improvement in hemolysis - The patient died at the age of 45 months of bronchopneumonia, pericardial effusion, and congestive heart failure
Case report of AIHA [33]	1; pediatric; M	- AIHA presenting with severe anemia - DAT: IgG+ C+. IAT positive as well - First-line treatment: prednisolone 45 mg/day, then IVIG 20 mg in association (CR) - Second-line treatment at reactivation one year later: rituximab 375 mg/m^2^ (four doses) and regular transfusion of red cell antigen (major and minor)-matched blood (CR)

RBC: red blood cells, autoAbs: autoantibodies; alloAbs: alloantibodies; AIHA: autoimmune hemolytic anemia; IV: intravenous; IVIG: intravenous immunoglobulin; F: female; M: male, CR: complete remission; PR: partial remission; DAT: direct antiglobulin test (with monospecific antisera unless otherwise specified).

**Table 3:** Studies and case series/reports about RBC autoAbs/AIHA in SCD and CHAs.

Type of Study Objective of the Study Disease	Number of pts; Pediatric/Adult; Sex	Main Findings
Prospective study SCD, TDT [27]	158 Pediatric and adult NA	- Freque;ncy of RBC autoAbs: 12% - DAT: C+ (*n* = 12) - The antibody did not appear to be clinically important since it became thermally reactive at 4 °C and not at 37 °C - Risk factors: alloAbs
Retrospective study SCD [28]	167 Pediatric and adult NA	- Frequency of RBC autoAbs: 3.6% of the total, 8–9.7% of the transfused - DAT: panagglutinin with no apparent specificity (83%) - Only 2/12 patients developed AIHA - Risk factors: alloAbs - Treatment and response of AIHA: - case 1 (IgG): steroids, IVIG, EPO, and RBC transfusions. CR after 2 months - case 2: unknown
Retrospective study. Frequency, serological characteristics, and clinical significance of autoimmunization in pediatric patients SCD [29]	184 Pediatric NA	- Frequency of RBC autoAbs: 8% - DAT: IgG+ (*n* = 9), IgG+ C+ (*n* = 5) - AIHA 4/14 patients (all IgG+ C+) - Risk factors: alloAbs - Treatment: corticosteroids, RBC transfusion - Response: one fatal hemolytic reaction following transfusion
SCD [30]	12 Pediatric and adult	- Frequency of RBC autoAbs: 42% - DAT: IgG+ (*n* = 1), C+ (*n* = 4) - Risk factors: alloAbs, chronic transfusion
Case series SCD [35]	5 Pediatric and adult F 2, M 3	- DAT: IgG+ C+ - Risk factors: alloAbs - Treatment: steroids; mercaptopurine in one patient - Response: CR/PR; autoAbs reverted to negative in all the patients after hospital discharge
Case series and review of the literature SCD [36]	2 Adult 1 M, 1 F	- Case 1: severe anemia (Hb 2.8 g/dL). Treated with prednisone, IVIG, and RBC transfusion - Case 2: severe anemia (Hb 4 g/dL), LDH was 3175 IU, reticulocytes 28%. IgG+. Treated with prednisone with CR
HS [11]	91 adult 40 F, 51 M	- Frequency of RBC autoAbs by mitogen-stimulated DAT: 61%—IgG fraction bound to α- and β-spectrin, Band 3, and Band 4.9 - The positive cases displayed increased reticulocytosis and slightly reduced hemoglobin (Hb) values compared to the negative ones

TDT: transfusion-dependent thalassemia, SCD: sickle cell disease; RBC: red blood cells, autoAbs: autoantibodies; alloAbs: alloantibodies; AIHA: autoimmune hemolytic anemia; IV: intravenous; IVIG: intravenous immunoglobulin; F: female; M: male, DAT: direct antiglobulin test (with monospecific antisera unless otherwise specified); EPO: erythropoietin; CR: complete remission; NA: not available.

## Data Availability

The data presented in this study are available in the article.
